# Estimation of amyloid aggregate sizes with semi-denaturing detergent agarose gel electrophoresis and its limitations

**DOI:** 10.1080/19336896.2020.1751574

**Published:** 2020-04-19

**Authors:** Polina B. Drozdova, Yury A. Barbitoff, Mikhail V. Belousov, Rostislav K. Skitchenko, Tatyana M. Rogoza, Jeremy Y. Leclercq, Andrey V. Kajava, Andrew G. Matveenko, Galina A. Zhouravleva, Stanislav A. Bondarev

**Affiliations:** a Department of Genetics and Biotechnology, St. Petersburg State University, St. Petersburg, Russia; b Institute of Biology, Irkutsk State University, Irkutsk, Russia; c Laboratory for Proteomics of Supra-Organismal Systems, All-Russia Research Institute for Agricultural Microbiology, St. Petersburg, Russia; d International Research Institute of Bioengineering, ITMO University, St. Petersburg, Russia; e Vavilov Institute of General Genetics Russian Academy of Sciences, St. Petersburg Branch, St. Petersburg, Russia; f Centre de Recherche En Biologie Cellulaire De Montpellier, UMR 5237 CNRS, Montpellier, France; g Laboratory of Amyloid Biology, St. Petersburg State University, St. Petersburg, Russia

**Keywords:** Amyloids, Sup35, Rnq1, SDD-AGE, Shiny application

## Abstract

Semi-denaturing detergent agarose gel electrophoresis (SDD-AGE) was proposed by Vitaly V. Kushnirov in the Michael D. Ter-Avanesyan’s laboratory as a method to compare sizes of amyloid aggregates. Currently, this method is widely used for amyloid investigation, but mostly as a qualitative approach. In this work, we assessed the possibilities and limitations of the quantitative analysis of amyloid aggregate size distribution using SDD-AGE results. For this purpose, we used aggregates of two well-characterized yeast amyloid-forming proteins, Sup35 and Rnq1, and developed a protocol to standardize image analysis and process the result. A detailed investigation of factors that may affect the results of SDD-AGE revealed that both the cell lysis method and electrophoresis conditions can substantially affect the estimation of aggregate size. Despite this, quantitative analysis of SDD-AGE results is possible when one needs to estimate and compare the size of aggregates on the same gel, or even in different experiments, if the experimental conditions are tightly controlled and additional standards are used.

## Introduction

Amyloids, fibrous protein aggregates with a set of specific features (resistance to protease or detergent treatment, cross-beta structure, and staining by Thioflavin T or Congo Red), are present in a variety of natural systems from bacteria to human. Different amyloid-forming proteins and the corresponding amyloid fibrils have very diverse functions and are implicated in a wide range of normal and pathological processes. For example, amyloids are important for transmission of information in yeast cells, memory formation in some animals, and development of neurodegenerative diseases in mammals (reviewed in [1–5]).

Size and other physical properties of amyloid aggregates are crucial features, as they frequently affect the fitness of cells. Small oligomers of several human amyloids can lead to cell death, but larger aggregates may have no effect on viability [6]. In the case of yeast prions, for example the [*PSI*^+^] factor, aggregate size is one of the distinctive features of the prion variants [7], and yeast strains propagating different [*PSI*^+^] variants also differ in their ability to suppress nonsense mutations [8].

There are few methods to estimate the size of amyloid fibrils formed in living cells, mainly size exclusion chromatography [9] and semi-denaturing detergent agarose gel electrophoresis (SDD-AGE) [7]. SDD-AGE, initially proposed by Vitaly V. Kushnirov in the Michael D. Ter-Avanesyan’s laboratory [7], is considered easier and less expensive. It is routinely used to screen for amyloid aggregates [10] but has also been applied for a more detailed analysis of the properties of these aggregates (reviewed in [2]). This method utilizes a characteristic property of amyloids, their resistance to detergents (mostly sodium dodecyl sulfate (SDS)), and is based on the ability of aggregates to enter agarose gels and distribute according to their molecular weight (strictly speaking, electrophoretic mobility). The latter allows us to suppose that SDD-AGE can be used for quantitative analysis of molecular weight distributions of amyloids *in vivo*. However, the possibility of such quantitative analysis has never been comprehensively evaluated.

In this work, we made an attempt to assess the possibilities and limitations of the quantitative analysis of amyloid aggregate size distribution using SDD-AGE results. In theory, such analysis would need (1) a tool for the transition from an image to intensity values, (2) a set of molecular weight standards, and (3) a tool for statistical comparison. The first problem can be solved with a number of packages, one of the most common being ImageJ/Fiji [11,12]. The second problem does not have a well-developed and commonly accepted solution, and such high molecular weight protein standards (MDa range) are not commercially available. Some authors have used high molecular weight proteins from animal muscles and commercial DNA molecular weight ladders as standards to estimate the molecular weight of the aggregates [7,13]. Finally, no specific user-friendly tools have been developed to draw statistically informed conclusions about the molecular weight distributions of molecules visualized with Western blotting, even though this problem can be easily solved in statistics-oriented programming environments such as R [14]. To this end, we developed a computational method to evaluate the parameters of amyloid aggregate size distribution depending on the DNA ladder. It allowed us to compare the aggregates sizes in different experimental conditions and investigate the possible limitations of SDD-AGE based quantitative analysis. We first of all tested the potential of SDD-AGE quantification on the example of Sup35 aggregates. This yeast release factor [15,16] forms amyloid aggregates, which are well-studied. The appearance and propagation of the [*PSI*^+^] prion depend on the aggregation of the Sup35 protein. Several well-characterized [*PSI*^+^] variants differ in the size of Sup35 aggregates (reviewed in [2]) and, thus, provide a good model for testing the approach. Apart from *in vivo* samples, we analysed *in vitro* generated amyloid aggregates of N-terminal part of Sup35 and full-length Rnq1 (the protein required for the [*PIN*^+^] prion propagation [17,18]) to compare molecular weights of aggregates estimated with SDD-AGE results and other methods.

## Materials and methods

### Yeast strains, media and growth conditions

*Saccharomyces cerevisiae* strains OT55 and OT56 [19,20] are isogenic derivatives of 74-D694 (*ade1-14 trp1-289 ura3-52 his3-∆200 leu2-3,112* [*psi*^−^][*PIN*^+^]) [21] with weak and strong [*PSI*^+^] variants, respectively. GT488 [22] and GT490 [23] are derivatives of OT55 and OT56, respectively. A [*PSI*^+^] strain with a different background, P-2V-P3982 (*МАТ*α *ade1-14 his7-1 lys2-87 ura3Δ leu2-B2 thr4-B15* [*PSI^+^*][*PIN*^+^]) [24], was also used. Yeast strains were cultured at 30°C in standard liquid and solid YEPD media [25]. Cells for further experiments were collected in the logarithmic phase of growth (optical density at 600 nm (OD_600_) = 0.6–0.8).

### Recombinant protein purification and in vitro fibril generation

For Sup35NM purification, the pET-20b-SUP35NM plasmid [26] was used. This vector contains a construct encoding Sup35NM fused to a His_6_-tag under control of an inducible T7 promoter. For purification of the Rnq1 protein, we constructed the pPROEX-HTb-RNQ1 plasmid by cloning the BamHI-SacI restriction fragment of pID129 [27] into the backbone of pPROEX-HTb-Sis1 [28] construct digested with the same pair of enzymes. For protein production, we used *Escherichia coli* strain BL21 (DE3) [29]. Cells were grown at 37°C in the 2TY with ampicillin (1.6% (w/v) tryptone, 1.0% (w/v) yeast extract, 5.0% (w/v) NaCl, 0.01% (w/v) ampicillin) medium. Isopropyl β-D-1-thiogalactopyranoside (IPTG) was added to the final concentration of 0.1 mM (for Sup35NM) or 1 mM (for Rnq1) to induce protein overproduction, and the cells were collected after 6 h of growth with IPTG.

Proteins were purified in denaturing conditions (in the presence of 8 M urea) according to previously published protocols [30]. A two-step purification procedure with Ni-NTA agarose (Invitrogen) and Q-sepharose (GE Healthcare) columns was performed for Sup35NM. Only the first step was used for Rnq1 purification. Proteins were concentrated using an Amicon Ultra Centrifugal filter (30 kDa, Millipore) or precipitated by adding five volumes of methanol and stored at −80°C. To obtain aggregates of Sup35NM, the protein was diluted at least 100-fold in the fibril assembly buffer (5 mM potassium phosphate, pH 7.4, containing 150 mM NaCl) to the final protein concentration of 0.5 mg/mL. Samples were incubated at 26°C with slow overhead rotation (Bio RS-24 rotator, Biosan) for 24 h. In these conditions, Sup35NM aggregates spontaneously. Rnq1 fibrils were generated according to the published protocol [31]. Briefly, the purified protein was dissolved in a buffer containing 4 M urea, 150 mM NaCl, and 5 mM potassium phosphate buffer (pH 7.4) and diluted to a final concentration of 0.5 mg/ml. The mixtures were then incubated at room temperature with rotation for 5–14 days. The formation of SDS-resistant aggregates was monitored with SDS-PAGE with boiled and unboiled samples [32]. Aggregates of both proteins bound the ThT dye. The resistance to limited proteolysis with PK, seeding and Congo Red staining were demonstrated for Sup35NM aggregates (data not shown).

### Transmission electron microscopy (TEM) imaging

For TEM measurements, we used formvar coated copper grids in conjunction with the method of negative staining with a 1% (w/v) solution of uranyl acetate . Jeol JEM-2100 and Jeol JEM-1400 (Japan) transmission electron microscopes were used. Fibrils lengths on TEM images were measured with ImageJ/Fiji software [11,12]. To analyse the effect of SDS treatment on the estimated fibril lengths, we added SDS to the fibril solutions to a final concentration of 2% (w/v) prior to sample preparation. Carbon coated grids were used for this experiment, as well as for all experiments with the Rnq1 fibrils.

### Protein extraction from yeast cells

Protein extraction with mechanical cell disruption (the B-method, from glass beads) was performed according to the published protocol [32] but with a modified lysis buffer (100 mM Tris-HCl pH 7.5, 50 mM NaCl, 10 mM 2-mercaptoethanol, 2% (v/v) protease inhibitor cocktail (Sigma, USA), 2 mM phenylmethylsulfonyl fluoride (PMSF)) according to the published protocol [33]. Cells were homogenized with a FastPrep24 benchtop homogenizer (MP Biomedicals, USA) at 6.0 M/S for 30 s and then incubated on ice for at least 30 s. The procedure was repeated 3 times. The B-method was used unless noted otherwise.

Protein extraction with spheroplasting (the S-method, from spheroplast) was performed according to the published protocol [33] with slight modifications. One of the two enzymes, lyticase (Sigma, USA) or zymolyase (MPI, USA) was used for spheroplasting. The cells were incubated in the enzyme solution at 30°C for 2–60 min and then collected by centrifugation at 800 rcf for 5 min at room temperature. The supernatant fraction was discarded and the pellet resuspended in the lysis buffer (see above). Then, the samples were homogenized with the FastPrep24 benchtop homogenizer (MP Biomedicals, USA) at 6.0 M/S for 30 s and incubated on ice for at least 30 s. The procedure was repeated 1–2 times. Finally, the samples obtained with either method were centrifuged at 4000 rcf for 2 min. The supernatant fraction was used in all further experiments.

### SDD-AGE

The SDD-AGE was performed according to the published protocol [7]. Gels with 1.5% (w/v) agarose were run at 30 V for 200–240 min in all experiments. Particular values of the parameters mentioned above are required to calculate a model describing the relationship between DNA size and mobility [34] and use it to estimate molecular weights of aggregates, so they should be recorded. Proteins were transferred onto a polyvinylidene fluoride (PVDF) membrane according to R. Halfmann and S. Lindquist's protocol [33]. Noteworthy, further analysis with *AGECalibratoR* requires precise alignment of membrane and gel edges. The DNA ladder (1 kb ladder, SibEnzyme, #M12) was prepared according to the standard procedure for protein samples [7]: mixed with the SDS-containing loading buffer and incubated for 5 min at room temperature. The TAE solution [35] was used as a running buffer (pH was adjusted with acetic acid).

For DNA staining, the corresponding part of the gel was cut off and placed into ethidium bromide solution for at least 1 h. A digital camera (Canon PowerShot G12) was used to obtain images. The rabbit polyclonal anti-Sup35 antibody SE4290 [36] and mouse monoclonal anti-His antibody (GE Healthcare, #27-4710-01) were used to detect Sup35 or His_6_ tag, respectively. The HRP conjugated secondary antibody (anti-mouse and anti-rabbit, GE Healthcare) was used for detection. Signals were recorded with the GeneGnome device (SynGene). The images of the stained DNA ladder and Western blotting signals were combined, scaled and aligned with the GIMP (gimp.org) software for further analysis in ImageJ/Fiji.

### Determination of spheroplasting efficiency

Spheroplasting efficiency was estimated by resuspending cells pelleted from 10 ml of initial suspension culture (OD_600_ = 0.6–0.8) in 1 ml of spheroplasting buffer [33]. An aliquot of this suspension was mixed with an equal volume of 1% (w/v) SDS, and the optical density (OD_600_) of this suspension was determined. This value was used as 100% OD. Then, we added lyticase to the solution and incubated the mix at 30°С. At each time point, an aliquot of this suspension was taken, mixed with an equal volume of 1% (w/v) SDS, and OD_600_ was recorded for these suspensions. These values were used to calculate relative values for each time point (% of the initial optical density for the corresponding sample). Spheroplast lysis triggered by SDS lowers both OD_600_ and cell concentration [37]. These data were fitted to the following model for the interaction between the relative OD_600_ and incubation time:

$$RelativeOD\;=\;a\;+\;b\;\times \;e^{\left(c\;\times \;time\right)}$$

### SDD-AGE based estimation of amyloid aggregate molecular weights

We proposed a two-step procedure for obtaining the quantitative estimates of the aggregate size distribution from SDD-AGE results. In the first step of the method, density plot profiles of western blotting signals are collected with ImageJ/Fiji [11,12] (here we used ImageJ [11] v.1.51). In the second step, a model describing the relationship between size and mobility of molecules [34] is calculated based on the DNA mobility. Given that the mobility of the ladder is independent of the experiment, we can extrapolate the model to protein aggregates, estimate their molecular weight and compare data from different experiments. For simplification of this step, we developed a tool *AGECalibratoR*, which is available online (https://bioinfo.crbm.cnrs.fr/index.php?route=tools&tool=26 and https://drozdovapb.shinyapps.io/AGECalibratoR_2/) and as a standalone application (the source code is deposited on https://github.com/drozdovapb/AGECalibratoR). For the estimation of aggregates molecular weights profiles corresponding to DNA ladder, background for Western blotting signal and the Western blotting signal are required. These data can be loaded into the graphical user interface of *AGECalibratoR*, which returns the arbitrary molecular weight of aggregates calculated according to the mobility of the DNA ladder on the same gel. The detailed user manual with a ready to use example is provided in the supplementary material.

Two different strategies of molecular weight estimation were implemented in the *AGECalibratoR*. The first is the median of the molecular weight distribution, and the second is the value corresponding to the main peak in the OD profile. The latter may provide more accurate results (more similar to the visible pattern as compared to the first metric) if signal to noise ratio is low. The high background signal adds noise to the data and can artificially shift the median value. However, it is worth noting that generally medians provide a narrower range of values obtained in different technical and biological replicates. Thus, we suggest medians as a metric of choice. *AGECalibratoR* was written with the Shiny with the following packages shinydashboard [38], DT [39], ggplot2, dplyr, tidyr [40], remotes [41], rPeaks [42], Rcpp [43], as well as coin [44] and multcomp [45] for statistical analysis.

### Statistical analysis

The R software environment [14] was used for the statistical analysis (Mann-Whitney rank sum test with FDR correction of the p-value) and plotting.

## Results and discussion

### The analysis of SDD-AGE reproducibility

**Figure 1.**
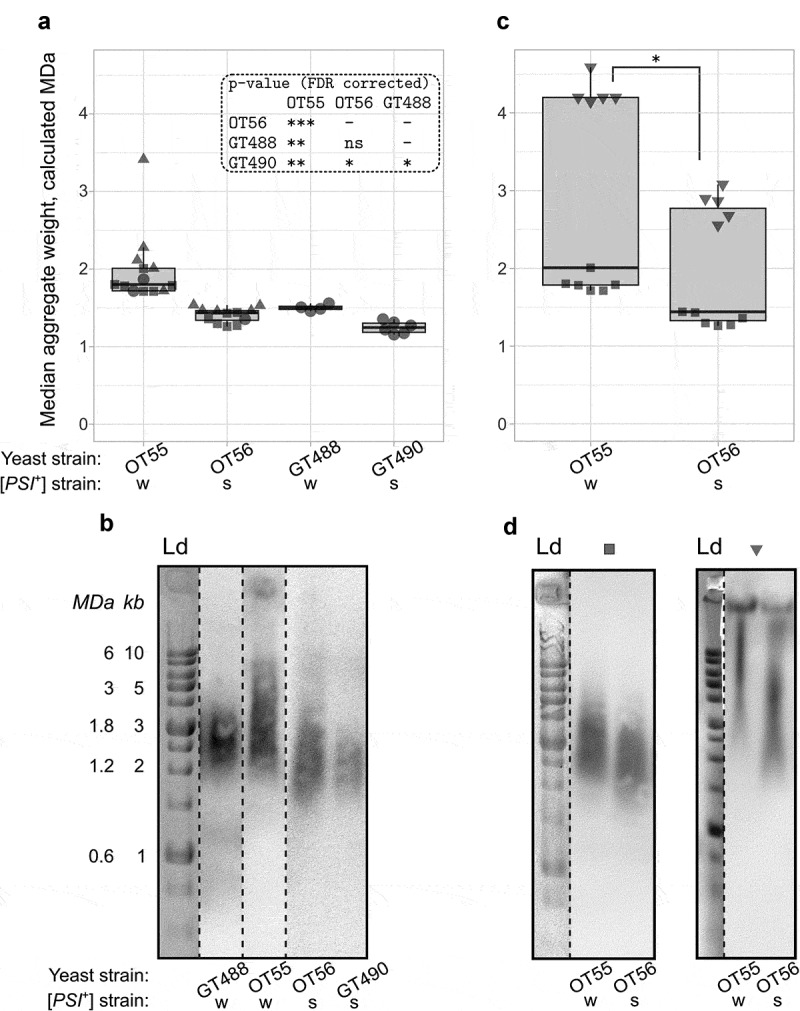
SDD-AGE can be used for quantitative analysis of aggregate molecular weights, but electrophoresis artefacts can make the results irreproducible. (a) The estimated median molecular weights of Sup35 aggregates in OT55 and GT488 (weak [PSI^+^]), as well as OT56 and GT490 (strong [PSI^+^]) yeast strains, demonstrate that the method can be used to compare aggregate sizes. ‘W’ and ‘S’ stand for weak and strong prion variants. (b) Representative images of SDD-AGE blots. The anti-Sup35 antibody was used. (c) Estimates of Sup35 aggregate sizes for two independent runs of different lysates of the OT55 (weak [PSI^+^]) and OT56 (strong [PSI^+^]) strains. Ld stands for the lane with DNA ladder. (d) Representative SDD-AGE images. Different dot shapes on the plots correspond to independent experiments. Additional lanes were removed for clarity (b, d). *, p < 0.05; **, p < 0.01; ***, p < 0.001, Mann-Whitney rank sum test, FDR-corrected).

The SDD-AGE method is an excellent tool to assess the presence of amyloid aggregates, but it is also used to estimate the distribution of their molecular weights. However, it is unclear whether accurate quantification and statistical comparison of these molecular weight distributions are possible. To test this, we reproduced the analysis of Sup35 aggregates in several yeast strains with the [*PSI*^+^] prion. The [*PSI^+^*] variants from the OT55 and OT56 strains are often considered as standard weak and strong prion variants. Previously, it was shown that they differ in Sup35 aggregates size [26]. We compared the results of five replicate SDD-AGE runs of OT55 and OT56 lysates (each time, a new sample of cells was used, in most cases these samples were collected from the same culture and should be considered as technical replicates). The estimation of molecular weights of these aggregates with *AGECalibratoR* statistically proved these differences and demonstrated that Sup35 aggregates in the weak variant were 25% larger than in the strong variant according to the median comparison. Moreover, the results of most independent SDD-AGE experiments were reproducible, since the estimates were very close to each other (Figure 1(a)). This fact in general demonstrated the accuracy of the proposed approach for quantification and usage of DNA ladder as a molecular weight marker for protein aggregates.

We also evaluated aggregate size parameters in two additional strains, GT488 and GT490, which were obtained as [*pin^−^*] derivatives of OT55 and OT56, respectively, by *HSP104^KT^* overexpression [22,23]. We found that Sup35 aggregates in GT488 were bigger than in GT490, and this difference was statistically significant. It also correlated with the phenotypes of these strains, as GT488 grew slower on the media selective for cells with the prion than GT490 (data not shown). Also, we hypothesized that Sup35 aggregates in these strains and their respective ancestors should be equal in size or at least comparable. Our data support this assumption, as for strains with strong [*PSI*^+^] variants, sizes of corresponding aggregates were close to each other, but statistical testing demonstrated the difference. For weak variants, the difference was more profound. Consistently, this difference could also be observed in the raw results of Western blotting (Figure 1(b)). These results might be explained by either analysis artefact or the real tiny difference linked to changes in the prion variants caused either by *HSP104^KT^* overexpression or by appearance of new mutations or spontaneous changes in the prion properties.

While investigating the effect of different factors on the results of SDD-AGE, we found that sometimes the results can vary unexpectedly for unknown reasons (for instance, see Figure 1(d)). Such artefacts caused large deviations in aggregate size estimates (Figure 1(c), downward facing triangles), even though the difference between OT55 and OT56 aggregate sizes remained significant (Figure 1(c)). Despite the fact that the potential irreproducibility of the SDD-AGE quantification in different experiments was revealed, we can at least suggest using the proposed method to quantify relative changes in the aggregates sizes on the same electrophoresis run. The approach might also be used for analysis of different experiments, but additional controls, allowing to prove the reproducibility of the electrophoresis conditions, are strongly recommended. For instance, the same sample can be loaded in all gels as an internal standard.

### The molecular weight of aggregates can only be estimated in arbitrary units

**Figure 2.**
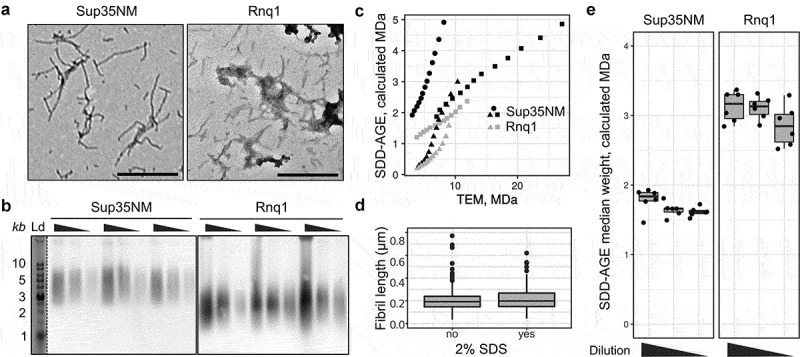
SDD-AGE-based estimates differ from the corresponding measurements based on the TEM data. (a) TEM images of Sup35NM and Rnq1 fibrils. The scale bar equals 500 nm. (b) SDD-AGE results for the same samples from panel A. The 3-fold serial dilutions were analysed. The anti-His antibody was used. ‘Ld’ corresponds to DNA ladder. (c) Quantile-quantile plots of aggregate molecular weights calculated with different methods. Percentiles from 20 to 80 in increments of 5 are plotted. Different dot shapes on the plots correspond to independent experiments. Different proteins are marked with colour (black for Sup35NM and grey for Rnq1). (d) The distributions of Rnq1 fibril lengths in samples with or without SDS. At least 250 fibrils were measured in each case. (e) The estimated median molecular weights of Rnq1 and Sup35NM fibrils computed using samples without or with 3-fold and 9-fold dilution. The corresponding raw results of SDD-AGE are shown in (b).

As our results demonstrate the possibility of quantitative comparison of aggregate molecular weight from SDD-AGE data under certain conditions, we next questioned whether the molecular weight estimates provided by *AGECalibratoR* are concordant with measurements made using other methods. DNA and protein molecules of the same nominal molecular weight have different charge and shape, and can thus migrate at slightly different speeds. The true correspondence between the mobility of DNA and protein fragments in SDD-AGE is (to the best of our knowledge) unknown. So, we tried to determine this correspondence empirically. To do so, we compared aggregate sizes estimated by *AGECalibratoR* with direct measurements of fibril size from TEM images. To this end, we used fibrils formed *in vitro* from purified recombinant Sup35NM and Rnq1 proteins (see the Materials and Methods section). The same fibril solutions were used for TEM and SDD-AGE analysis (Figure 2(a,b)). We used TEM results to measure the lengths of several hundred representative fibrils and calculate the expected molecular weight of each fibril using a reference value of fibril unit length of 0.47 nm/monomer (0.47 nm is a distance between protein molecules in fibril with cross-beta structure (see review [46])). We then calculated the quantiles of the resulting fibril molecular weight distribution and compared these values with the matching ones provided by *AGECalibratoR* analysis of SDD-AGE gels. This analysis revealed that the results obtained by two measurement methods show notable differences (Figure 2(c)) and were different between replicates.

This observation might either reflect inherent differences in the migration rates of DNA and protein molecules in SDD-AGE or indicate that SDS treatment affects the size distribution of amyloid aggregates analysed using TEM. To test the latter hypothesis, we analysed how treatment of fibril solutions with 2% SDS prior to imaging affects the estimated length of amyloid fibrils. We found no differences in the length of Rnq1 fibrils after treating them with SDS, indicating that SDS treatment does not explain the differences between SDD-AGE and TEM results (Figure 2(d)). Thus, the observed discordance is most likely to be caused by differences in DNA and protein mobility, making it hard to make precise estimates of fibril molecular weight from SDD-AGE data. We also questioned whether the amount of protein loaded into the gel affects the result of molecular weight estimation. To address this question, we compared the estimated median molecular weights of Rnq1 and Sup35NM fibrils after 3-fold and 9-fold dilution. As shown in Figure 2(e), aggregate molecular weights calculated using diluted samples were slightly lower, though the difference had no statistical significance in the Wilcoxon test. These results suggest that protein concentrations should be carefully adjusted before loading the samples onto SDD-AGE gel for adequate quantitative comparison.

We also hypothesized that the observed variations between experiments (Figure 2) can reflect differences in fibril samples. For the presented analysis we assumed that fibrils revealed by TEM included only one protofibril and have one protein molecule in the cross-section. This idea is in agreement with several works on Sup35 fibrils [47–49], but data from several other works may support other hypotheses [50]. Sup35 fibrils may contain several protofibrils and, thus have several protein molecules in cross-section [50]. Moreover, it is unknown which types of aggregates can be revealed with SDD-AGE. Our analysis assumed that we observe separate amyloid fibrils on the SDD-AGE results, we cannot exclude that actually clumps of protein aggregates are observed. Thus, the aforementioned results lead to the conclusion that the aggregate mass can only be estimated in arbitrary molecular weight units (called ‘calculated MDa’ in *AGECalibratoR*) and these measurements should be interpreted in terms of numbers of molecules per aggregate with great care. Nevertheless, these arbitrary units leave a possibility to calculate relative changes in aggregate size.

### Running buffer pH affects the mobility of amyloid aggregates

**Figure 3.**
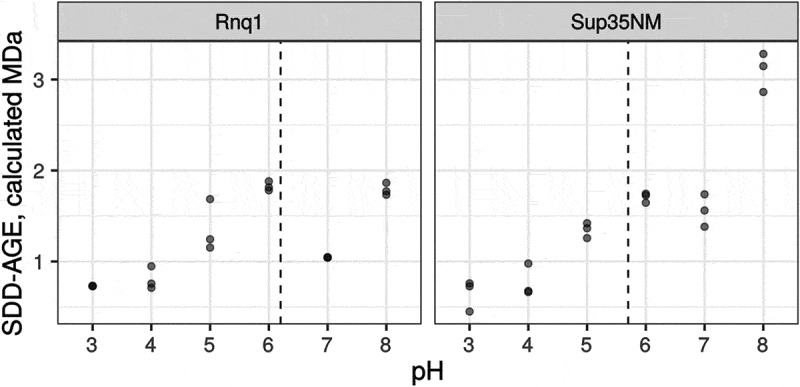
pH of the running buffer affects the mobility of amyloid aggregates. Each dot corresponds to the median molecular weight of Rnq1 or Sup35NM fibrils obtained *in* *vitro* estimated with SDD-AGE in running buffers with the respective pH. Results of 3 replicate runs are shown. Dashed lines correspond to isoelectric points of proteins.

Yet another factor that may influence the reproducibility of SDD-AGE results and the concordance between TEM and AGE estimates is the pH of the running buffer. The charge of the protein molecules affects their mobility in the gel. The SDD-AGE protocol assumes that SDS in the loading and gel running buffers unify the charge of all the protein molecules. However, the effect of buffer pH on the mobility of amyloid aggregates has never been studied. To address this issue, we analysed the mobility of Sup35NM and Rnq1 aggregates obtained *in vitro* in running buffers with different pH values. These two proteins have different isoelectric points, 5.7 and 6.2, respectively (calculated with the ExPASy web service [51]). Our results demonstrated that the pH value modified the mobility of aggregates in a protein-specific manner (Figure 3). In our experiments, the estimated size of Sup35NM aggregates varied over a wider range compared to Rnq1. This may be linked to the clusters of charged amino acid residues present in the M domain of Sup35, which play a role of pH sensors and regulate the formation of biomolecular condensates under acidic stress [52]. Notably, such clusters are absent in the Rnq1 protein.

### Cell lysis technique affects the mobility of amyloid aggregates

**Figure 4.**
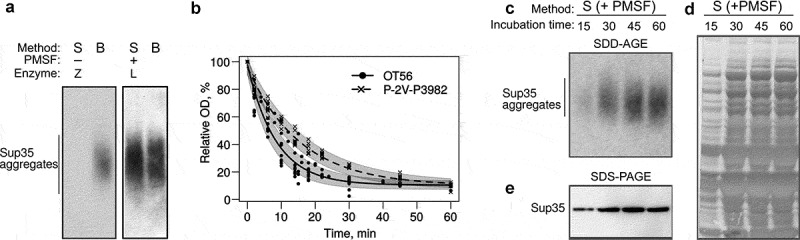
Optimization of spheroplast lysis. (a) The comparison of the protein extraction methods before (left) and after (right) optimization of spheroplast lysis. Zymolyase 20T and lyticase 2M (indicated as Z and L, respectively) were used for spheroplasting to create images in the left and right parts of the panel, respectively. The anti-Sup35 antibody was used. (b) Dynamics of spheroplasting measured by OD. (c-e) Analysis of protein extracts at different time points. Panels (a)and (c)show SDD-AGE results, panel (d)shows a Coomassie R250 stained polyacrylamide gel, and E shows Western blotting after SDS-PAGE. The extracts of the OT56 strain were used in panels (a)and (c-e).

The original protocol of the SDD-AGE [7] was adopted for high throughput screening in the Susan L. Lindquist’s laboratory [33]. The major modification introduced into the new protocol touched on a cell lysis step. There are two main techniques for protein extraction from yeast cells in non-denaturing conditions, which are necessary to study amyloid aggregates. The first method is based on mechanical cell disruption with glass beads [32], heretofore the B-method, while the second utilizes enzymatic disruption of cell walls, i.e. spheroplasting [33], heretofore the S-method. To compare the two methods, we reproduced the original protocol for the S-method and applied it to the OT56 strain. Cells were collected at the late logarithmic phase, pelleted and stored at −80°C until use, but no longer than for 30 days. The obtained extracts were analysed with SDD-AGE. The same cells were processed with the B-method [32]. Surprisingly, we could only detect Sup35 aggregates in the extracts obtained with the B-method (Figure 4(a), left). Thus, we obtained proof that the method of cell lysis can affect the results of SDD-AGE.

To overcome this limitation, we decided to optimize protein extraction conditions and checked whether contaminants in the enzyme can influence the quality of the protein extract. The original protocol [33] utilized zymolyase produced by Seikagaku Corporation (Japan), which contained 1 × 10^5^ units of zymolyase per 1 g and 0.17 units of protease per 1 unit of zymolyase. The enzyme available to us (zymolyase by MPI, USA, heretofore zymolyase 20T) contained 2 × 10^4^ units per 1 g and 0.5 protease units per 1 zymolyase unit, which could definitely negatively influence the quality of protein extracts. We tried replacing zymolyase 20T with lyticase (Sigma, USA; heretofore lyticase 2M). The manufacturer does not indicate protease activity of this enzyme, but its activity is much higher than that of zymolyase 20T and constitutes 2 × 10^6^ units per 1 g, so we can assume lower protease activity in the final solution. We compared the efficiency of protein extraction using the S-method with either zymolyase 20T and lyticase 2M, but this did not completely prevent the proteolysis, compared to the B-method (data not shown). Then we tried adding PMSF (phenylmethylsulfonyl fluoride), which acts as a serine protease inhibitor, to the spheroplasting buffer. PMSF is known to slow down proteolysis during spheroplasting [53]. Similar to the previous experiment, we used protein extracts obtained with the B-method as a control. We found that the addition of PMSF indeed prevented the proteolysis, as we obtained the same results for both methods (Figure 4(a), right).

To further improve the method and possibly shorten the procedure, we analysed how spheroplasting efficiency depended on the incubation time in the lyticase solution. For this, we used two [*PSI*^+^] strains, ОТ56 and P-2V-P3982, which differ by their ability to form cell clumps in the suspension culture due to mutations in *AMN1* and *FLO8* [54]. This parameter should influence the spheroplasting efficiency. Indeed, in the case of the less clumping-prone strain OT56 the OD values dropped down to 25% within the first 15 min of incubation, reached their minimum at 30 min and almost did not change later (Figure 4(b)). In contrast, the clumping-prone strain Р-2V-P3982 reached its minimal OD value only at 45 min. These results suggest that clumping hinders the action of lyticase. However, we cannot rule out the possibility that some other differences in the genotype of the studied strains also contribute to spheroplasting efficiency. Moreover, we used SDD-AGE to analyse the extracts of OT56 cells treated with lyticase for different times. This experiment showed that 15 min of incubation was enough to detect Sup35 aggregates (Figure 4(c)). However, it is worth noticing that longer incubation times (30 min) increased signal strength in terms of aggregate detection (Figure 4(c)), the total amount of detectable Sup35 (Figure 4(e)) and total protein amount in the sample (Figure 4(d)). These parameters did not change much during further incubation (Figure 4(c–e)), which correlates with the changes in optical density (Figure 4(b), solid line).

**Figure 5.**
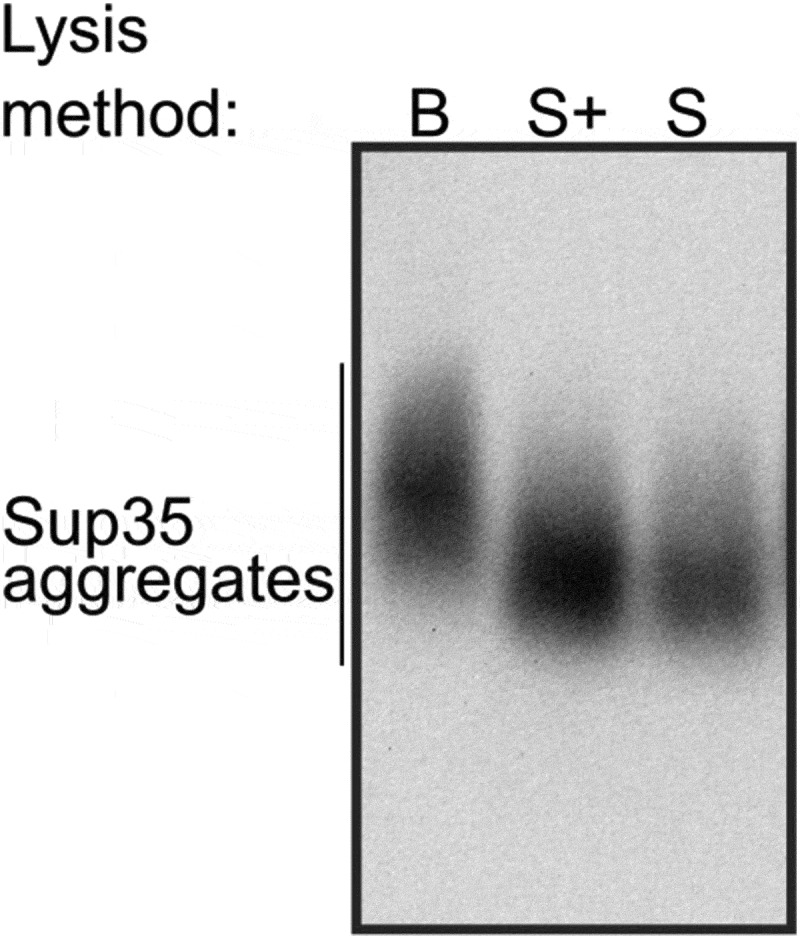
*The cell lysis technique may affect the size of Sup35 aggregates*. B, cell lysis with beads, S, spheroplast lysis, S+, modified spheroplast lysis with PMSF added to the spheroplasting buffer. The OT56 strain was used for this experiment.

Finally, we reproduced the result with another batch of lyticase 2M for OT56. In this case, we worked with cells which were not frozen (dissimilar to the previous experiments) and found no difference between extracts with and without PMSF added at the spheroplasting stage but found a surprising differenсe between molecular weights of Sup35 aggregates in the extracts obtained with the B- and S-methods (Figure 5). We suppose that the Sup35 aggregates extracted with the S-method may become lighter due to molecular chaperone-mediated cleavage, activated under the stress conditions (digestion of the cell wall). The published data on transcriptional changes in *S. cerevisiae* triggered by zymolyase treatment [55] showed that the expression of *SSA3, SSA4, HSP104,* and *SIS1* genes is 1.5 to 2-fold higher in this condition. Lyticase treatment is likely to cause a similar response. The disbalance between the components of chaperone machinery was shown to destabilize the [*PSI*^+^] prion [22]. These data are clearly insufficient to pinpoint the exact mechanism of the possible shift in aggregate size, but we suggest being careful when comparing the results obtained with different lysis methods and only compare sizes of samples obtained with the same method whenever possible.

## Conclusions

Here we describe possibilities and limitations of quantitative estimation of amyloid aggregate sizes based on SDD-AGE. Importantly, we showed that the mobility of aggregates and their estimated sizes depend on multiple factors, including the chosen cell lysis technique, pH of the gel running buffer, and any artefacts distorting the signal. Recent work also revealed that the size of Sup35 aggregates is changing upon culture growth [56]. Taken together, these facts demonstrate the severe limitations of the SDD-AGE results quantification. However, we show that quantitative analysis is still possible, at least to compare the size of aggregates on the same gel. Our new approach, *AGECalibratoR*, allows this but only in arbitrary units, which do not correspond to daltons. The estimation can then be used to calculate the relative changes in aggregate sizes. We also showed that reproducible results can be obtained in different experiments if the experimental conditions are tightly controlled and additional standards are used.
